# miR-148b-3p functions as a tumor suppressor in GISTs by directly targeting KIT

**DOI:** 10.1186/s12964-018-0228-z

**Published:** 2018-04-16

**Authors:** Yu Wang, Jun Li, Dong Kuang, Xiaoyan Wang, Yuanli Zhu, Sanpeng Xu, Yaobing Chen, Henghui Cheng, Qiu Zhao, Yaqi Duan, Guoping Wang

**Affiliations:** 1 Institute of Pathology, Tongji Hospital, Tongji Medical College, Huazhong University of Science and Technology, 1095 Jiefang Dadao, Wuhan, 430030 People’s Republic of China; 2 Department of Pathology, School of Basic Medicine, Tongji Medical College, Huazhong University of Science and Technology, Wuhan, 430030 People’s Republic of China; 3 Department of Gastroenterology, Zhongnan Hospital, Wuhan University, Wuhan, 430071 People’s Republic of China

**Keywords:** Gastrointestinal stromal tumor (GIST), KIT, miRNA, miR-148b-3p

## Abstract

**Background:**

Gain-of-function mutations and overexpression of KIT are characteristic features of gastrointestinal stromal tumor (GIST). Dysregulation in miRNA expression may lead to KIT overexpression and tumorigenesis.

**Methods:**

miRNA microarray analysis and real-time PCR were used to determine the miRNA expression profiles in a cohort of 69 clinical samples including 50 CD117^IHC+^/KIT^mutation^ GISTs and 19 CD117^IHC−^/wild-type GISTs. GO enrichment and KEGG pathway analyses were performed to reveal the predicted targets of the dysregulated miRNAs. Of the dysregulated miRNAs whose expression was inversely correlated with that of KIT miRNAs were predicted by bioinformatics analysis and confirmed by luciferase reporter assay. Cell counting kit-8 (CCK-8) and flow cytometry were used to measure the cell proliferation, cycle arrest and apoptosis. Wound healing and transwell assays were used to evaluate migration and invasion. A xenograft BALB/c nude mouse model was applied to investigate the tumorigenesis in vivo. Western blot and qRT-PCR were used to investigate the protein and mRNA levels of KIT and its downstream effectors including ERK, AKT and STAT3.

**Results:**

Of the six miRNAs whose expression was inversely correlated with that of KIT, we found that miR-148b-3p was significantly downregulated in the CD117^IHC+^/KIT^mutation^ GIST cohort. This miRNA was subsequently found to inhibit proliferation, migration and invasion of GIST882 cells. Mechanistically, miR-148b-3p was shown to regulate KIT expression through directly binding to the 3’-UTR of the KIT mRNA. Restoration of miR-148b-3p expression in GIST882 cells led to reduced expression of KIT and the downstream effectors proteins ERK, AKT and STAT3. However, overexpression of KIT reversed the inhibitory effect of miR-148b-3p on cell proliferation, migration and invasion. Furthermore, we found that reduced miR-148b-3p expression correlated with poor overall survival (OS) and disease-free survival (DFS) in GIST patients.

**Conclusion:**

miR-148b-3p functions as an important regulator of KIT expression and a potential prognostic biomarker for GISTs.

## Background

GIST is the most common mesenchymal tumors of the digestive tract, representing 1–3% of gastrointestinal malignancies [1]. Activating mutations in the receptor tyrosine kinase KIT or platelet-derived growth factor receptor alpha (PDGFRA) are the principal oncogenic triggers of GIST, which have been identified in ~ 80% and 5–10% of GISTs, respectively [2]. Approximately 10% of GIST cases do not contain KIT and PDGFRA mutations, which are called ‘wild-type’ (wt) GISTs [3, 4]. Recent studies have revealed that wt GISTs represent a heterogeneous group in clinical, pathological and biological features that is profoundly different from KIT/PDGFRA mutant tumors [5]. Moreover, the wt GISTs often exhibit a greater degree of resistance to the tyrosine kinase inhibitor imatinib [6].

MicroRNAs (miRNAs) comprise a class of 18–25 nucleotides, non-coding RNAs that specifically bind to the 3′-untranslated region (3’-UTR) of target mRNAs [7, 8]. Growing evidence indicates that miRNAs are involved in multiple cancer-relevant processes, including migration, invasion and angiogenesis [9–11]. To date, several studies have explored the function of miRNAs in the tumorigenesis of GISTs, demonstrating the involvement of miRNA dysregulation in gene mutation, chromosomal changes, tumor risk and metastasis [12–15]. Since activating mutation in the KIT gene is the main oncogenic trigger in > 80% of GISTs, the aberrant expression of miRNAs that interrelates with KIT-induced tumorigenesis is of particular interest [13].

With this in mind, we profiled for miRNA expression in a cohort of 5 CD117^IHC+^/KIT^mutation^ and 5 CD117^IHC−^/wild-type GISTs and validated our findings in another cohort of 59 samples comprising 45 CD117^IHC+^/KIT^mutant^ and 14 CD117^IHC**−**^/wild-type GISTs. By this approach, we identified 6 miRNAs that were dysregulated and whose expression was inversely related to KIT mutation or KIT overexpression. In particular, miR-148b-3p emerged as a significantly downregulated miRNA that we subsequently showed to suppress proliferation, migration and invasion of GIST882 cells by directly targeting the 3′-UTR of KIT at nucleotides 1639–1656. Restoration of miR-148b-3p expression in GIST882 cells led to reduced expression of not only KIT but also its downstream effectors, including ERK, AKT and STAT3. Overexpression of KIT reversed the suppressive actions of miR-148b-3p on cell proliferation, migration and invasion. Moreover, the reduction of miR-148b-3p was correlated with high risk grade, high mitotic rate and the presence of recurrence or metastasis in GIST patients. Kaplan-Meier analysis indicated that GIST patients with low levels of miR-148b-3p correlated with poor overall survival (OS) and disease-free survival (DFS) rates. These results indicate that miR-148b-3p plays an essential role in promoting the progression of GISTs by reducing KIT expression. Furthermore, our work has identified miR-148b-3p as a potential prognostic biomarker for GIST and a candidate therapeutic target.

## Methods

### Patients and tissue samples

Formalin-fixed paraffin-embedded (FFPE) samples of 115 primary GISTs, including 80 cases of CD117^IHC+^ GISTs and 35 cases of CD117^IHC-^ GISTs, were selected from over 1000 GIST patients who received surgery at Tongji Hospital between January 2007 and December 2012 (Table 1). No patient received receptor tyrosine kinase inhibitor therapy before the surgery. All were assessed for clinical and histomorphological features, and the risk grade was evaluated on the basis of the National Institute of Health (NIH) consensus classification criteria. Ethical approval was obtained from the Ethics committee of Tongji Hospital, Tongji Medical College, Huazhong University of Science and Technology. Each participant provided written informed consent to join this study.

**Table 1:** Clinical characteristics of GIST samples included in this study

Characteristics	No. of patients (%)
Age (years)	
≤ 55	68 (59.1)
> 55	47 (40.9)
Gender	
Male	63 (54.8)
Female	52 (45.2)
Primer sites	
Stomach	51 (44.3)
Small instine	31 (27.0)
Colon	15 (13.0)
EGIST	18 (15.7)
Tumor size (cm)	
≤ 5	41 (35.7)
> 5 to ≤10	49 (42.6)
> 10	25 (21.7)
Histological type	
Spindle phenotype	95 (82.6)
Epithelioid phenotype	11 (9.6)
Mixed phenotype	9 (7.8)
Mitotic rate (per 50 HPFs)	
≤ 5	65 (56.5)
6 to 10	27 (23.5)
> 10	23 (20.0)
National Institutes of Health risk group	
Very low risk	9 (7.8)
Low risk	34 (29.6)
Intermediate risk	39 (33.9)
High risk	33 (28.7)
CD117	
Postive	80 (69.6)
Negative	35 (30.4)
Relapse	
Yes	12 (10.4)
No	103 (89.6)

All cases had been confirmed by immunohistochemical and KIT and PDGFRA mutational analyses before diagnosis. Immunohistochemical staining was performed on FFPE specimens using EnVision systems (Dako). The QIAamp DNA FFPE Tissue Kit (56,404; Qiagen; Hilden, Germany) was applied to isolate genomic DNA from FFPE tissues. KIT exons 9, 11, 13, 17 and PDGFRA exons 12, 18 were amplified with specific primers (Table 2). Additionally, for the 24 cases of GISTs lacking mutations, immunohistochemical analysis was carried out to measure the expression of succinate dehydrogenase complex subunits B (SDHB) in order to detect SDH-deficiency GIST. The 19 cases of CD117^IHC−^/wild-type GISTs employed for microarray and qRT-PCR analysis were all positive for DOG1 and SDHB.

**Table 2:** Association between miR-148b-3p expression and clinicopathological parameters in 59 GIST patients

Characteristics	Number	Percent	Median exprssion of miR-148b-3p/U6	*P* value
Age (years)				
≤ 55	37	62.71	1.49 ± 0.79	0.54
> 55	22	37.29	1.36 ± 0.72	
Gender				
Male	32	54.24	1.35 ± 0.79	0.33
Female	27	45.76	1.55 ± 0.73	
Primer sites				
Stomach	21	35.59	1.59 ± 0.69	0.38
Small instine	22	37.29	1.28 ± 0.68	
Others	16	27.12	1.36 ± 0.82	
Tumor size (cm)				
≤ 5	30	50.85	1.74 ± 0.63	0.07
> 5 to ≤10	19	32.20	1.19 ± 0.81	
> 10	10	16.95	1.03 ± 0.77	
Histological type				
Spindle phenotype	54	91.53	1.46 ± 0.76	0.85
Epithelioid phenotype	2	3.39	1.38 ± 0.91	
Mixed phenotype	3	5.08	1.20 ± 1.13	
Mitotic rate (per 50 HPFs)				
≤ 5	32	54.24	1.91 ± 0.61	< 0.001
6 to 10	20	33.90	0.85 ± 0.45	
> 10	7	11.86	0.98 ± 0.74	
National Institutes of Health risk group				
Very low to low risk	21	35.60	2.11 ± 0.30	< 0.001
Intermediate risk	19	32.20	1.26 ± 0.71	
High risk	19	32.20	0.88 ± 0.64	
Relapse				
Yes	10	18.64	0.80 ± 0.41	0.04
No	49	81.36	1.58 ± 0.77	

### MicroRNA profiling

The miRNA microarray profiling was conducted by LC Sciences (Houston, TX) according to Sanger miRBase Version 19.0. Hybridization was carried out on a μParaflo microfluidic chip (Atactic Technologies, Houston, TX, USA). Fluorescent signals were scanned by a microarray scanner (GenePix 4000B, Molecular Device, USA) and analyzed using the GenePix Pro 6.0 software. The Locally Weighted Regression by LOWESS filter was used to normalize the data. The *P*-value was corrected by the false discovery rate (FDR). Only dysregulated miRNAs that fulfilled the benchmarks of *p* value < 0.05 and fold change > 1.5 were chosen out for further investigation. The microarray data have been submitted to the Gene Expression Omnibus (GEO) and the GEO accession number is GSE73346.

### Antibodies and reagents

The antibodies against SDHB (10620–1-AP, Proteintech, Philadelphia, PA, USA), ERK (9102, Cell Signaling, San Jose, CA, USA), phospho-ERK (4376, Cell Signaling), AKT (4691, Cell Signaling), phospho-AKT (4060, Cell Signaling), STAT3 (sc-482, Santa Cruz, Dallas, TX, USA), phospho-STAT3 (sc-8059, Santa Cruz), β-actin (sc-47,778, Santa Cruz), KIT (SAB4501647, Sigma-Aldrich, St Louis, MO, USA), HRP (horseradish peroxidase)-labeled anti-rabbit IgG (A0208, Beyotime Biotechnology, Shanghai, China), and HRP-labeled anti-mouse IgG (A0216, Beyotime Biotechnology) were used at the appropriate concentration.

### Oligonucleotides, plasmid construction and cell transfection

miR-148b-3p mimics, miR-148b-3p inhibitor, mimic control and inhibitor control were obtained RiboBio Co. (Guangzhou, Guangdong, China). KIT-coding sequences without the 3′-UTR were cloned into the GV141 vector by GeneChem (Shanghai, China) (called pGV141-KIT). The psiCHECK-2 vector (C8021) was purchased from Promega (Madison, WI, USA). The psiCHECK-2-KIT-wt, psiCHECK-2-KIT-mut-1378, and psiCHECK-2-KIT-mut-1639 were constructed by Bios Biological (Wuhan, Hubei, China). miR-148b-3p agomir and miR agomir NC were generated by GenePharma Co., Ltd. (Shanghai, China). Lipofectamine 3000 (L3000015, Invitrogen, Grand Island, NY, USA) was used to transfect cells with the desired genes or plasmids.

### Quantitative RT-PCR

Total RNA was reverse-transcribed by RevertAid cDNA Synthesis Kit (K1622, Thermo Fisher Scientific, Waltham, MA, USA) and amplified on an ABI Prism 7900HT platform (Applied Biosystems, Foster City, CA, USA) with specific miRNA primers (Table 2). U6 snRNA or GAPDH was used as endogenous control. The comparative Ct method was employed to estimate the relative expression levels.

### Target prediction, pathway and microRNA gene network analysis

The prediction of miRNA targets and the effector pathways were revealed by Gene Ontology (GO) enrichment [16] and Kyoto Encyclopedia of Genes and Genomes (KEGG) pathway analyses [17]. GO and KEGG terms with corrected *P*-value and FDR below 0.01 were defined as dramatically enriched in target candidate genes and pathways. By bioinformatics algorithms TargetScan and miRanda, we obtained the relationships between microRNAs and the predicted target genes from the gene databases. Interaction pattern between miRNAs and target genes were created using CytoScape [18].

### Cell culture

The GIST882 cell line with a KIT homozygous missense mutation (exon 13, K642E) was kindly provided by Dr. Jonathan Fletcher, Dana-Farber Cancer Institute, Boston, MA, USA. GIST882 cells were cultivated in RPMI-1640 (GIBCO, Carlsbad, CA, USA) containing 10% FBS (GIBCO) and cultured in a humidified incubator with 5% CO_2_ at 37 °C.

### Cell viability and proliferation assay

The Cell Counting Kit-8 kit (CCK-8; CK04–100, Dojindo, Kumamoto Prefecture, Kyushu, Japan) was used to evaluate cell viability. GIST882 cells (5 × 10^3^ cells/well) with or without imatinib treatment were placed into a 96-well plate and treated with 10 μl CCK8 solution for 3 h. Absorbance at 450 nm was detected by an ELx800 Absorbance Reader (BioTek Instruments, Inc., Winooski, VT, USA).

### Cell cycle and apoptosis analyses

The propidium iodide cell cycle analysis kit (C1052, Beyotime Institute of Biotechnology) was used to assess the cell cycle arrest of GIST882 cells on a FACScan flow cytometer (BD Biosciences, USA). The Annexin V-FITC/PI Apoptosis Detection Kit (556,547, BD Biosciences) was used to detect the apoptosis in GIST882 cells by flow cytometry.

### Cell migration and invasion assays

GIST882 cells suspended in RPMI-1640 medium containing 2% FBS were plated in the upper chamber to assess the migration ability, whereas cells suspended in serum-free RPMI-1640 were added into transwell inserts (3422, Corning, Bedford, MA, USA) precoated with Matrigel (356,234, Corning) to detect the invasion ability. The number of cells crossed over the membrane were fixed in 4% paraformaldehyde, dyed with 0.1% crystal violet and calculated under an inverted microscope after 24 h of incubation.

### Wound healing assay

Linear wounds were produced in the cell monolayer by a sterile pipette tip after cells cultures reached a confluence of 90–95%. The cells migrated into the wounded area or protruded from the border of the wound were quantified under an inverted microscope after 24 h of incubation in serum-free medium.

### Dual-luciferase reporter assay

The miR-148b-3p mimics and vectors (psiCHECK-2-KIT-wt, psiCHECK-2-KIT-mut-1378, or psiCHECK-2-KIT-mut-1639) were co-transfected into the GSIST882 cells via Lipofectamine 3000. The dual-luciferase reporter assay system (E1910, Promega) were conducted to assess the firefly and Renilla luciferase activities. Renilla luciferase was considered as an internal control.

### Western blot

Protein samples were prepared using RIPA lysis buffer (R0278, Sigma-Aldrich), phosphatase inhibitor cocktail tablets (04906845001, Roche) and protease inhibitor cocktails tablets (04693132001, Roche). Total protein was resolved on a 12% SDS-PAGE gel and transferred onto a nitrocellulose membrane. After blocking with 5% BSA, the membranes were blotted with primary antibodies overnight and secondary antibodies for 1 h. The SuperSignal West Femto Maximum Sensitivity Substrate (34,095, Thermo Fisher Scientific, Waltham, MA, USA) with HRP Substrate (34,095, Thermo Fisher Scientific) was employed to visualize the blots.

### Xenografts in nude mice

Athymic BALB/c nude mice (female, 4–6 weeks old) were provided by Huafukang Biotechnology Co. Ltd. (Beijing, China). All animal studies were approved by the Animal.

Ethics Committee of Tongji Hospital, Tongji Medical College, Huazhong University of Science and Technology. Approximately 5 × 10^6^ GIST882 cells suspended in culture medium were subcutaneously injected into the flanks of mice. Tumor growth was assessed biweekly and estimated using the formula: 1/2 (length × width^2^). When the tumor volume reached 0.5–0.6 cm^3^, miR-148b-3p agomir or miR agomir NC was syringe-injected into the tumor mass at the dose of 50 μg per mouse every week. After 3 weeks, the mice were euthanized and tumor tissues were made into paraffin sections for further study.

### Statistical analysis

The statistical analysis was implemented by the SPSS version 19.0 software. Student’s t-test was utilized to compare the variables between two groups, while one-way ANOVA was utilized to compare the variables among multiple groups. Fisher’s exact test and Chi-square test were applied to sort the GO and KEGG pathway categories. Kaplan-Meier method was performed to estimate OS and DFS and the log-rank test was carried out to assess the statistical difference between groups. Data were presented as mean ± SD from three independent experiments. *P* < 0.05 was considered statistically significant.

## Results

### MicroRNA profiling of GIST tissue

**Fig. 1:**
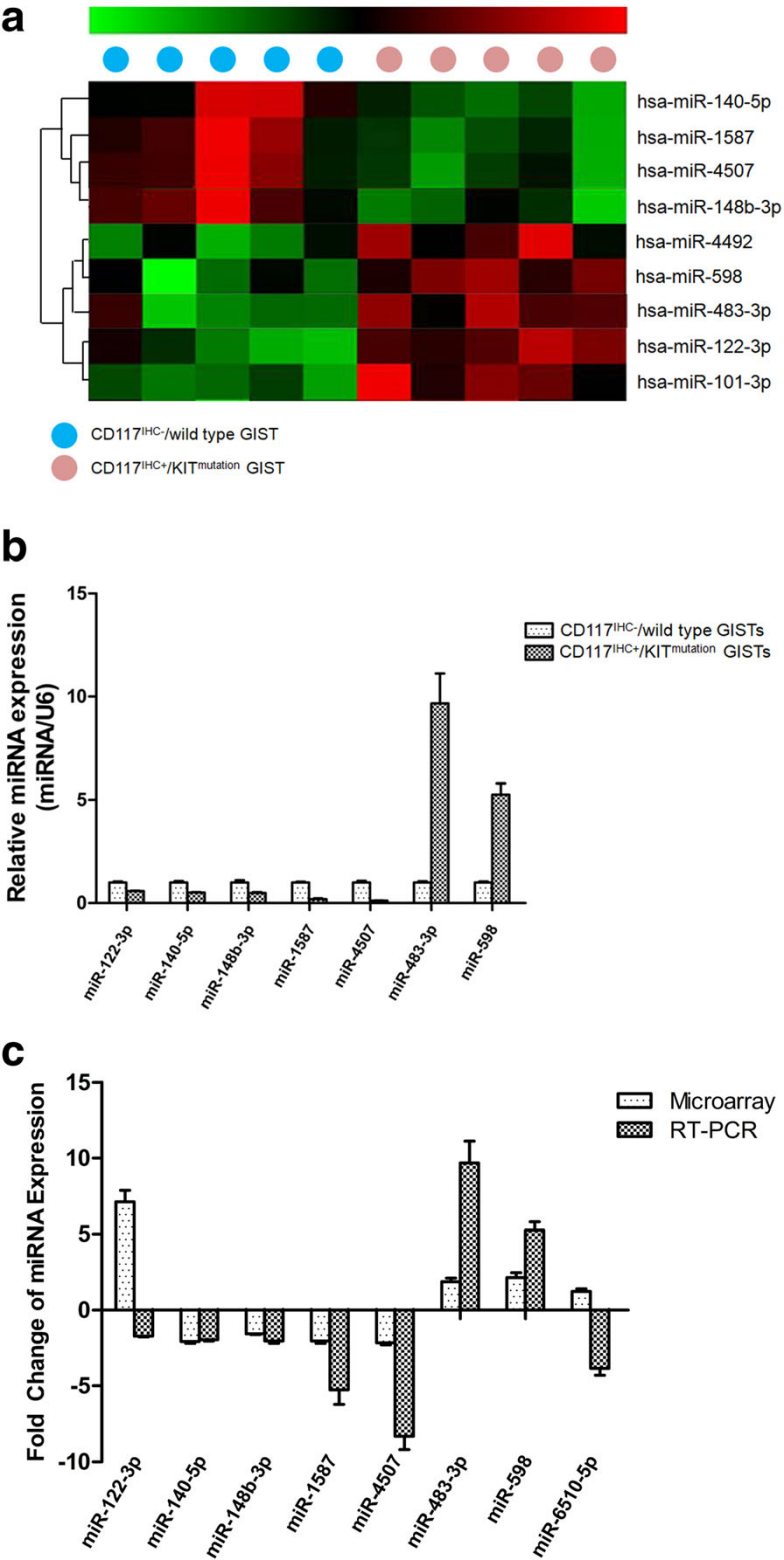
Differentially expressed miRNAs in CD117IHC+/KIT mutation GIST compared with CD117IHC-/wild type GIST patients. (**a**) A heat map based on miRNA microarray data that shows distinct expression patterns for 9 miRNAs between the two patient groups (fold change>1.5; p<0.05). (**b**) Validation of miRNA expression by RT-PCR in CD117IHC+/KITmutation GISTs compared to CD117IHC-/wild type GISTs. (**c**) A comparison between the microarray and RT-PCR data in the expression (fold change) for a group of miRNAs

The microarray data revealed distinct expression patterns for 9 mRNAs that were significantly different between the CD117^IHC+^/KIT^mutation^ GIST and CD117^IHC−^/wild-type GIST patient groups (fold change> 1.5; P < 0.05). Among these miRNAs, miR-122-3p, miR-483-3p, miR-101-3p, miR-598 and miR-4492 were significantly upregulated, whereas miR-140-5p, miR-148b-3p, miR-1587 and miR-4507 were significantly downregulated in the CD117^IHC+^/KIT^mutation^ GISTs. In complementary experiments, we used real-time PCR to determine changes in expression for 7 of these miRNAs between the two patient groups (Fig. 1b). Except for one, six miRNAs exhibited the same trend in both the microarray and RT-PCR analysis (Fig. 1c). These included four downregulated miRNAs (miR-140-5p, miR-148b-3p, miR-1587 and miR-4507) and two upregulated miRNAs (miR-483-3p and miR-598).

### Target prediction and miRNA-mRNA interaction network analysis

**Fig. 2:**
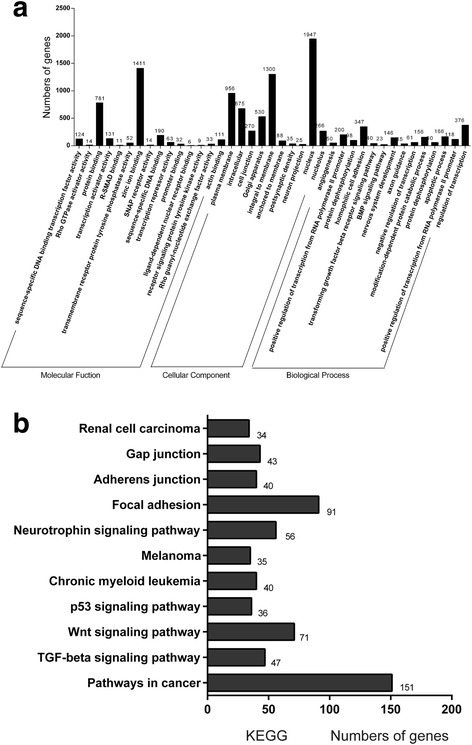
GO enrichment and KEGG pathway analyses. (**a**) GO analyses of the predicted target genes by molecular function, cellular component and biological process. (**b**) The KEGG pathway analyses of the commonly dysregulated genes

**Fig. 3:**
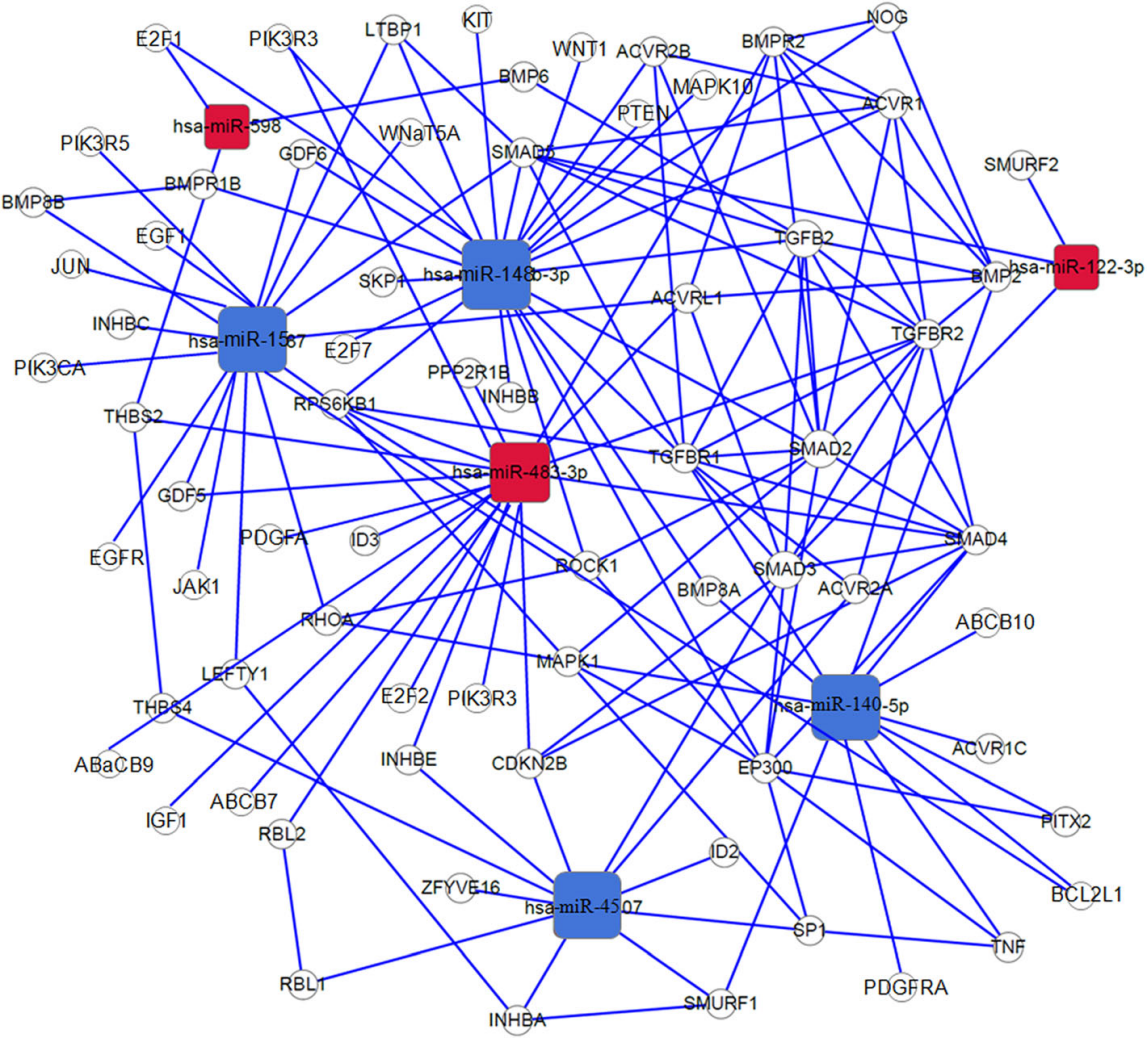
A network of the dysregulated miRNAs and their predicted target genes. Red/blue, miRNAup/down-regulated in CD117IHC+/KITmutation GISTs compared to CD117IHC-/wild type GISTs

TargetScan, miRBase and miRanda algorithms were employed to predict the target genes for the above six miRNAs (Table 3). GO enrichment and KEGG pathway analyses were subsequently performed to evaluate the functions of the target genes. The target genes were analyzed by GO enrichment analysis in molecular function, cellular component and biological process (Fig. 2a). KEGG pathway analysis revealed that 16,353 target genes were involved in 108 biological functions (Fig. 2b). A number of oncogenes, including KIT, PDGFRA, PIK3CA, AKT2, FGFR1, SMAD3, PTEN and EGFR emerged from both the GO and KEGG analysis of the miRNA-regulated gene network (Fig. 3). Because these genes have been shown to played essential roles in GIST tumorigenesis [19–21], our miRNA-mRNA regulatory network analysis revealed several candidate for future investigation of miRNA-target functions that control the development and progression of GIST.

**Table 3:** Predicted and validated targets for selected miRNAs

miRNA	Predicted targets	Valiadated targets in GIST tumorgenesis
miR-122-3p	40	SMAD3,STAT3
miR-140-5p	185	PDGFRA
miR-148b-3p	269	KIT,SMAD3,TP53,PTEN,AKT2
miR-1587	326	PIK3CA,EGFR
miR-4507	212	CDKN2A
miR-483-3p	208	FGFR1,NTRK3,CXCR4
miR-598	44	E2F1

### Identification of KIT as a direct target of miR-148b-3p through integrative bioinformatics analysis

Because of the pivotal role of KIT in GIST pathogenensis, we next focused our study on identifying the miRNA that regulates KIT expression in GIST. To this end, we performed a computational target prediction analysis by the TargetScan and miRanda bioinformatic algorithms in combination to search for miRNAs that may bind the 3′UTR of the KIT transcript. Of the six candidate miRNAs, we found that only miR-148b-3p could directly target KIT mRNA by interacting with the 3′-UTR of KIT at nucleotides 1378–1393 and 1639–1656 (Fig. 7a).

### miR-148b-3p suppressed the proliferation of GIST cells

**Fig. 4:**
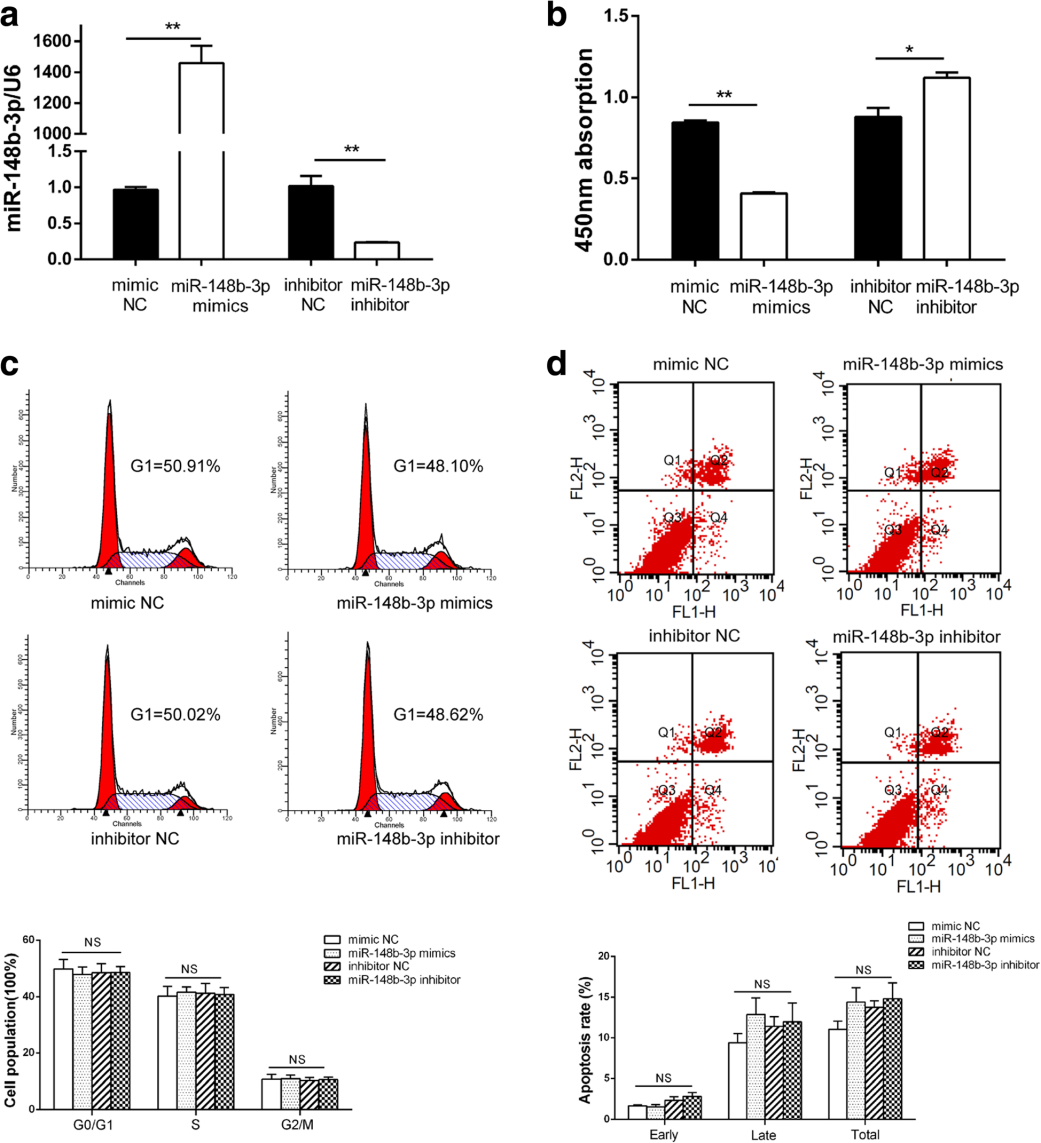
miR-148b-3p suppressed cell proliferation but had no effect on cell cycle and apoptosis in GIST882 cells. (**a**) RT-PCR was used to determine the transfection efficiencies of miR-148b-3p mimics or miR-148b-3p inhibitor in the GIST882 cells. (**b**) CCK8 assay was used to evaluate cell proliferation. (**c**) The cell cycle status was assessed by flow cytometry. (**d**) The cell apoptosis was evaluated by Annexin V-FITC/PI staining. *P* value was calculated by one-way ANOVA. *P<0.05, **P<0.01, NS non-significant

The biological significance of miR-148b-3p was assessed by transfecting the miR-148b-3p mimics, miR-148b-3p inhibitor, or the corresponding negative control into the GIST882 cells. The effects of the transfection were verified by qRT-PCR, which indicated that the mimic and inhibitor oligos affected miR-148b-3p expression in GIST882 cells as predicted (Fig. 4a). As shown by the CCK-8 assay, upregulation of miR-148b-3p by the mimics suppressed the proliferation of the GIST882 cells whereas downregulation of miR-148b-3p by the inhibitor oligo had an opposite effect (Fig. 4b). Intriguingly, neither the mimics nor the inhibitor had any effect on cell cycle arrest and apoptosis (Fig. 4c-d). These results indicate that miR-148b-3p has an anti-proliferative effect on the GIST882 cells.

### miR-148b-3p augmented the effect of imatinib in suppressing GIST cells proliferation

**Fig. 5:**
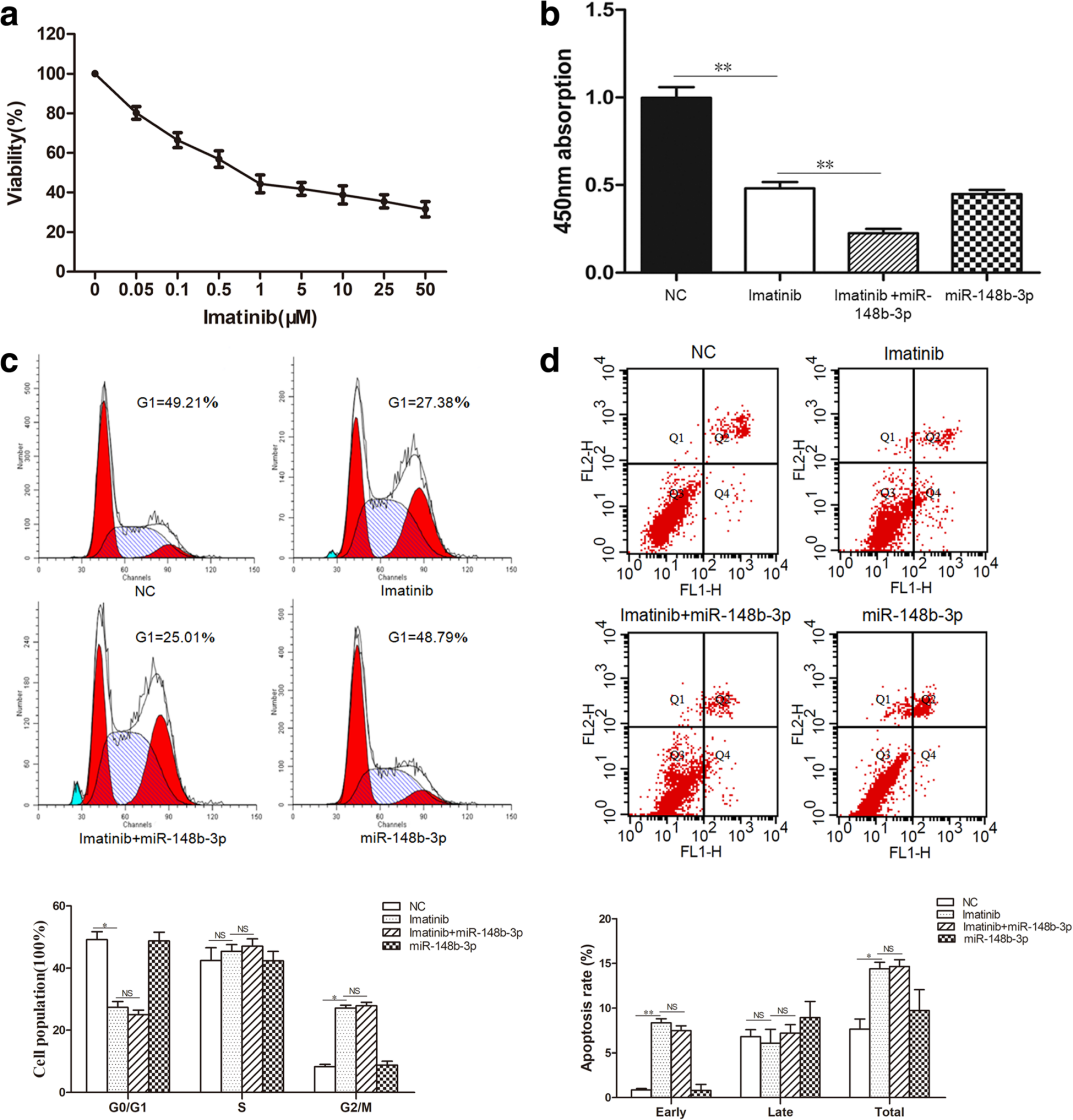
miR-148b-3p augmented the effect of imatinib in suppressing GIST cells proliferation. miR-148b-3p mimics was transfected into GIST882 cells after a 24 h treatment of 5 μM imatinib. (**a**) The IC50 value of GIST882 cells were measured after treatment with different concentrations of imatinib (0-50 μM) for 24h by cell viability assay. (**b**) CCK8 assay was used to evaluate cell proliferation. (**c**) The cell cycle status was assessed by flow cytometry. (**d**) The cell apoptosis was evaluated by Annexin V-FITC/PI staining. *P<0.05, **P<0.01, NS non-significant

To investigate whether miR-148b-3p sensitized GIST882 cells to imatinib, we firstly detected the IC_50_ concentration of imatinib using cell viability assay and confirmed that the IC_50_ values for imatinib in GIST882 cells was 1.654 μM for 24 h (Fig. 5a). We then evaluated the effect of miR-148b-3p in combination with a 24 h treatment of imatinib (5 μM) on proliferation, cycle arrest and apoptosis in GIST882 cells. As the data shown, imatinib treatment repressed cell proliferation and induced G1-phase arrest and apoptosis in GIST882 cells compared to untreated cells (Fig. 5b-d). Overexpression of miR-148b-3p in combination with imatinib treatment more notably inhibited cell proliferation in GIST882 cells compared to the cells treated with imatinib alone (Fig. 5b). However, miR-148b-3p overexpression did not affect cell cycle arrest and apoptosis in GIST882 cells either alone or in combination with imatinib treatment (Fig. 5c-d). These results indicated that miR-148b-3p acts synergistically with imatinib to suppress cell proliferation and sensitizes GIST882 cells to imatinib treatment.

### miR-148b-3p inhibited the migration and invasion of GIST cells

**Fig. 6:**
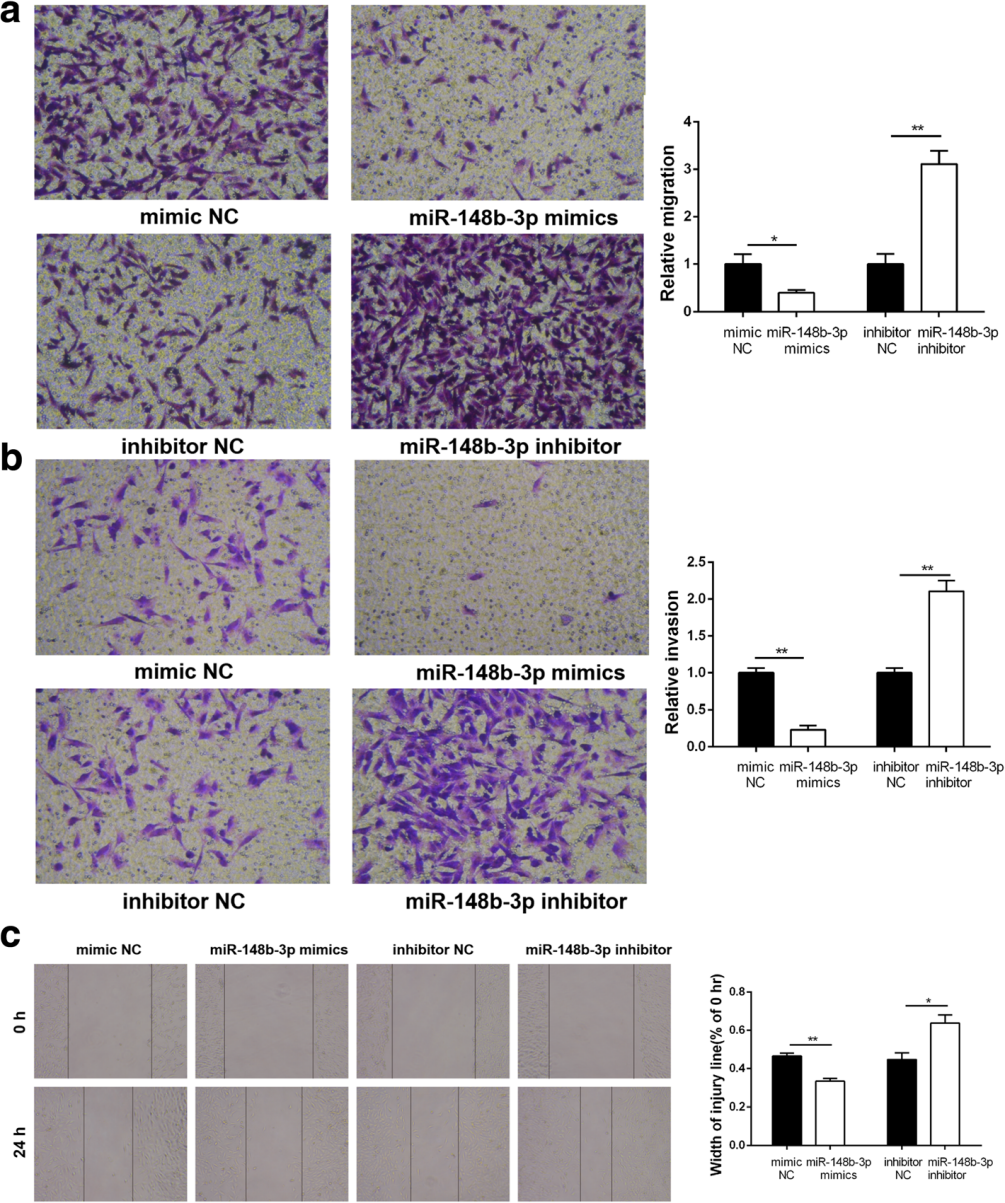
miR-148b-3p suppresses migration and invasion of GIST882 cells. (**a**) Transwell migration assay was applied to assess the migratory capacities of GIST882 cells. (**b**) Invasionassay was applied to detect the invasive capacities of GIST882 cells. (**c**) Wound healing assay was carried out to investigate the migratory ability of GIST882 cells. *P<0.05, **P<0.01

Microscopic observations on wound healing demonstrated that miR-148b-3p overexpression dramatically reduced the speed of wound closure in GIST882 cells (*P* < 0.01; Fig. 6c). In contrast, the wound closure speed was improved after miR-148b-3p inhibitor transfection (*P* < 0.05; Fig. 6c). Similarly, transwell migration and invasion assays showed that the cells penetrating the transwell membrane were markedly reduced in the miR-148b-3p mimics group (*P* < 0.05 and *P* < 0.01; Fig. 6a-b). In contrast, the miR-148b-3p inhibitor group exhibited high rates of mobility and invasiveness (*P* < 0.01; Fig. 6a-b). Collectively, miR-148b-3p upregulation markedly reduced the migration and invasion ability of GIST882 cells. This observation may have relevance to the progression and metastasis of GIST.

### KIT is a direct target of miR-148b-3p in GIST

**Fig. 7:**
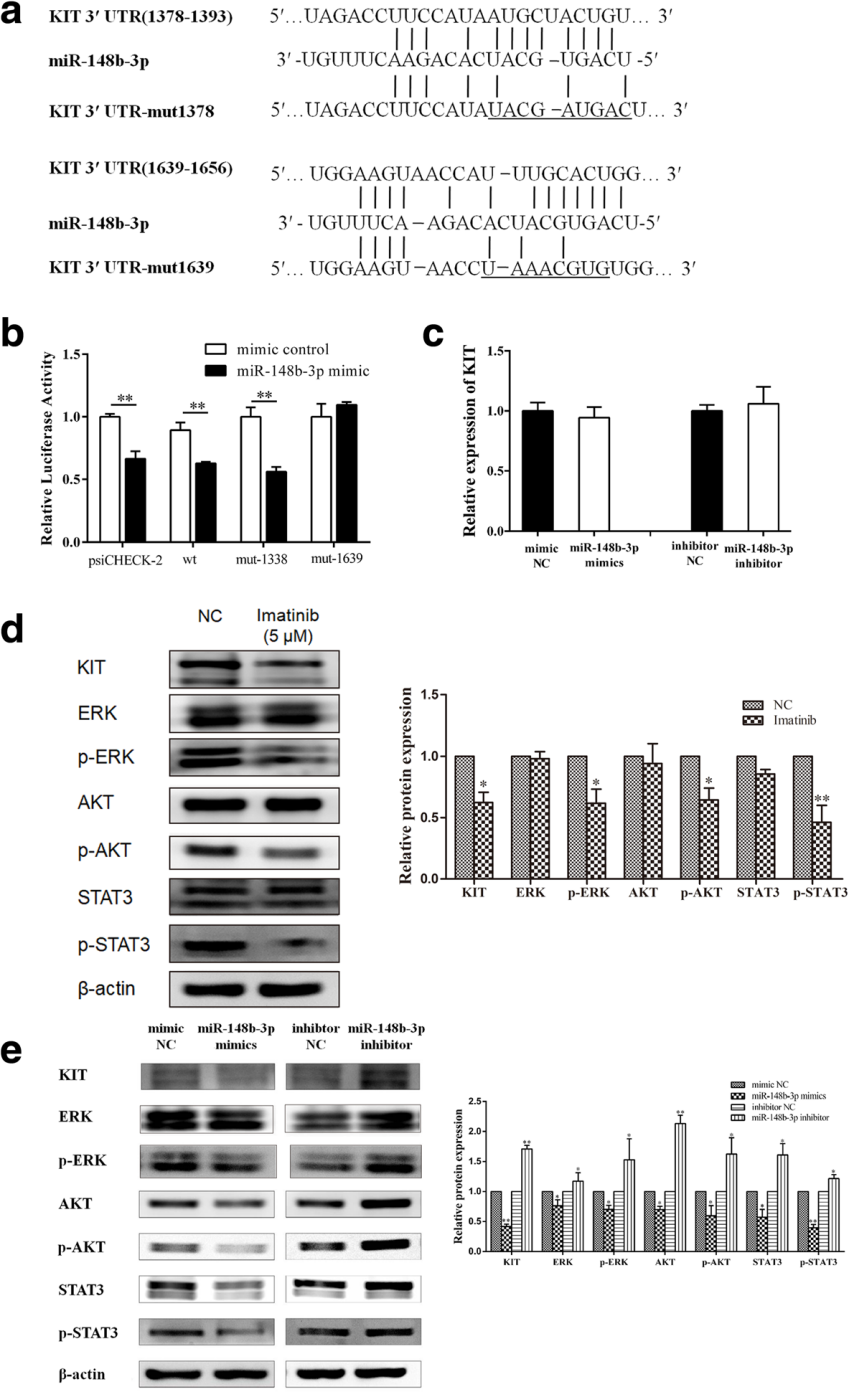
miR-148b-3p suppressed the expression of KIT and its downstream molecules ERK, AKT and STAT3 in GIST cells. (**a**) Bioinformatics analyses showed that miR-148b-3p potentially targeted KIT at two different sites. (**b**) Luciferase reporter assay identified the binding site of miR-148b-3p on the 3′-UTR of KIT in GIST882 cells. (**c**) KIT mRNA expression in GIST882 cells was evaluated by qRT-PCR analyses. (**d**) The protein expression of phosphorylated and total ERK, AKT and STAT3 in GIST882 cells treated with 5 μM imatinib were detected by western blot analyses. (**e**) The level of phosphorylated and total ERK, AKT and STAT3 in GIST882 cells with miR-148b-3p mimics or inhibitor transfection was respectively assessed by western blot analyses. *P<0.05, **P<0.01

To establish the miRNA-mRNA relations between miR-148b-3p and KIT, a dual-luciferase detection system was performed to measure the transcriptional activity of the KIT constructs after psiCHECK-2-KIT-wt or psiCHECK-2-KIT-mut transfection. As shown in Fig. 7b, miR-148b-3p mimics remarkably attenuated the relative luciferase activity of psiCHECK-2-KIT-wt and psiCHECK-2-KIT-mut1378 compared to the negative control. However, the miR-148b-3p mimics did not affect the relative luciferase activity of the psiCHECK-2-KIT-mut1639, suggesting that the sequence between nucleotides 1639–1656 is the major binding site of miR-148b-3p. Western blotting showed that transfection with miR-148b-3p mimics result in an obvious reduction of KIT protein, whereas that with miR-148b-3p inhibitor led to an increase in KIT in GIST882 cells (Fig. 7e). However, qRT-PCR indicated that miR-148b-3p alterations did not change the expression of endogenous KIT mRNA in GIST882 cells (Fig. 7c). Taken together, miR-148b-3p could directly target KIT mRNA in GIST882 cells by interacting with the 3′-UTR of KIT, and negatively regulated KIT protein expression by inhibiting the translation of KIT at the post-transcriptional level rather than mRNA degradation as they did not change KIT mRNA expression level.

### Downregulation of KIT leads to the inhibition of ERK, AKT and STAT3

To test whether the downregulation of KIT can lead to the inhibition of known signaling mediators downstream of KIT, we treated the GIST882 cells with 5 μM imatinib mesylate (Novartis) for 24 h and determined the protein levels of ERK, AKT and STAT3 by Western blot. As expected, the inhibition of KIT led to a rapid inhibition of phosphorylated ERK, AKT and STAT3 protein in GIST882 cells (Fig. 7d). These results implied that ERK, AKT and STAT3 activation is coupled to KIT activation in the GIST882 cells.

### KIT signaling is perturbed by miR-148b-3p mimics or inhibitor transfection

Transfection with miR-148b-3p mimics dramatically reduced phosphorylated and total ERK, AKT and STAT3 protein of GIST882 cells, while miR-148b-3p inhibition have the opposite effect (Fig. 7e). These results suggest that the oncogenic KIT signal transduction was modulated by miR-148b-3p in GISTs.

### Overexpression of KIT reversed the suppressor effects of miR-148b-3p on proliferation, migration and invasion of GIST cells

**Fig. 8:**
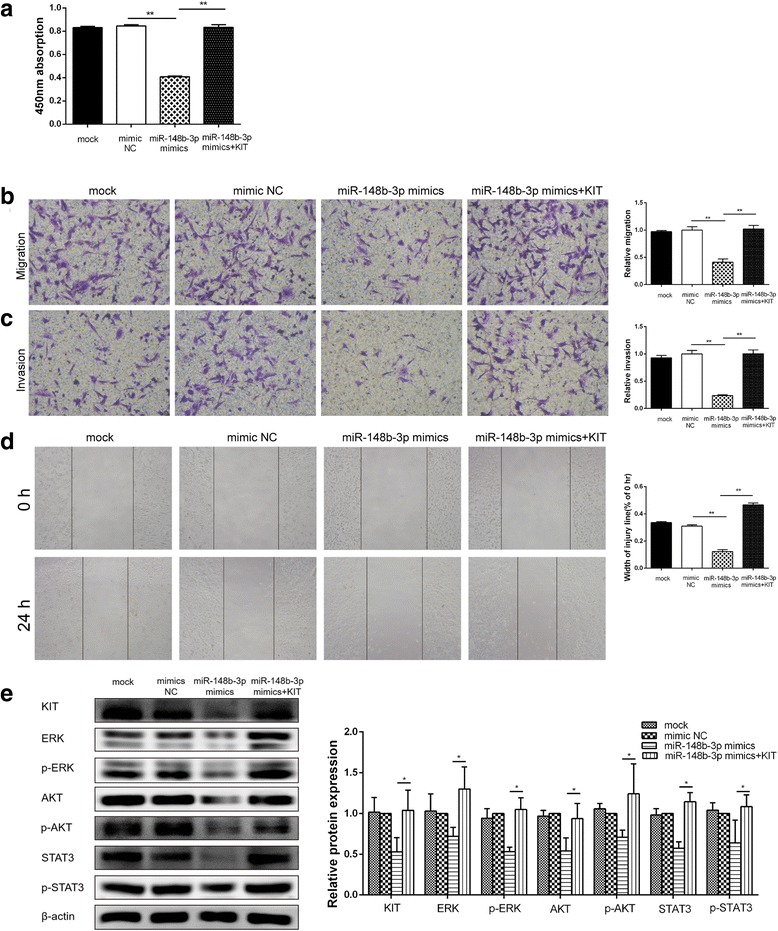
Overexpression of KIT reversed the inhibitory effect of miR-148b-3p on the proliferation, migration and invasion of GIST cells. (**a**) GIST882 cells co-transfected with pcDNA3.1-KIT and miR-148b-3p were measured for cell proliferation by the CCK8 assay. (**b**) The migratory capacities of GIST882 cells co-transfected with pcDNA3.1-KIT and miR-148b-3p were assessed by transwell migration assay. (**c**) The invasive capacities of GIST882 cells co-transfected with pcDNA3.1-KIT and miR-148b-3p were determined by invasion chamber assay. (**d**) The migratory ability of GIST882 cells co-transfected with pcDNA3.1-KIT and miR-148b-3p were investigated by wound healing assay. (**e**) The protein expression of phosphorylated and total ERK, AKT and STAT3 in GIST882 cells was assessed by western blot analyses. *P<0.05, **P<0.01

To elucidate whether the suppressive effects of miR-148b-3p on GISTs were induced by KIT repression, KIT expression was restored in GIST882 cells by transfecting the cells with the pGV141-KIT vector after miR-148b-3p mimics transfection. The ectopic overexpression of KIT not only rescued the cell growth inhibition induced by miR-148b-3p but also reversed the suppressor effectors of miR-148b-3p on the migration and invasion of GIST882 cells (Fig. 8a-d). Moreover, miR-148b-3p decreased the expression of phosphorylated and total ERK, AKT and STAT3 in GIST882 cells, all of which could be restored by KIT overexpression (Fig. 8e). These results suggest that KIT was directly responsible for modulating the biological effects of GIST cells induced by miR-148b-3p.

### miR-148b-3p suppresses tumor growth in vivo

**Fig. 9:**
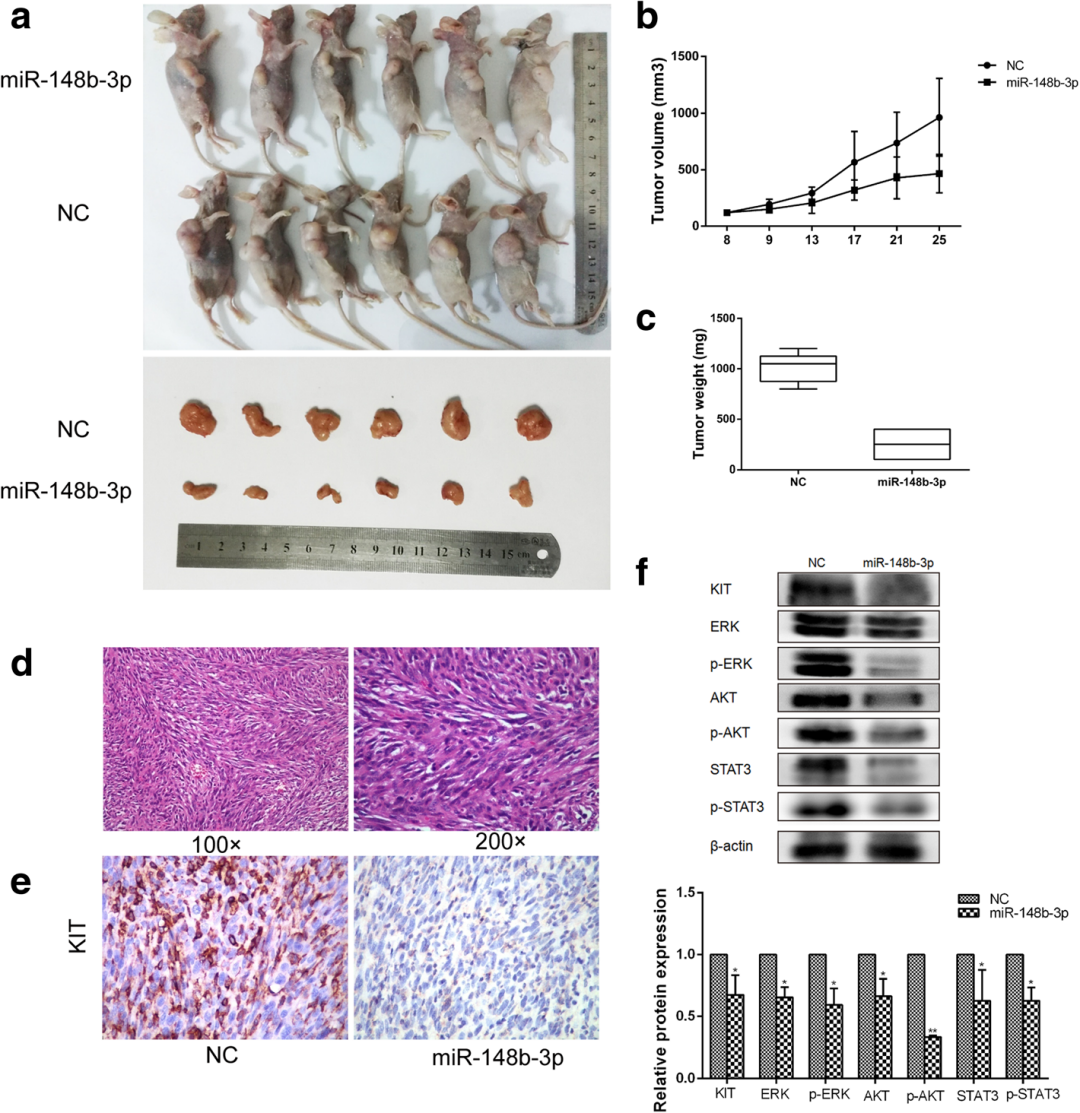
miR-148b-3p suppresses the growth of GIST in vivo. (**a**) Representative images of subcutaneous tumors in mice receiving the miR-148b-3p agomir or the control (NC). (**b**) Tumor growth curves for the group of mice receiving miR-148b-3p agomir compared with the control group. (**c**) Tumor weights for mice with miR-148b-3p agomir injection compared to the control. (**d**) Representative Hematoxylin and eosin (HE) staining pictures of subcutaneous tumors for mice treated with miR-148b-3p or the NC. (**e**) Representative immunohistochemical staining of subcutaneous tumors for mice treated with miR-148b-3p or the NC. (**f**) Western blot analyses of phosphorylated and total ERK, AKT and STAT3 protein expression in subcutaneous tumors. *P<0.05, **P<0.01

Consistent with its effects in vitro, miR-148b-3p remarkably suppressed the growth of xenografted tumors in mice. The tumors treated with miR-148b-3p agomir had a lower volume and weight than the tumors treated with miR agomir NC (Fig. 9b-c). Morphological similarities between the xenografts and human GISTs were evident based on stained paraffin-embedded sections (Fig. 9d). Immunohistochemical staining of the xenograft tumor sections indicated that the subcutaneous tumors of the miR-148b-3p-agomir-treated nude mice exhibited reduced signal for KIT compared to the control tumors (Fig. 9e). In parallel, Western blotting demonstrated that the tumors from the miR-148b-3p-agomir-treated mice exhibited a lower protein level of KIT and a significant reduction in phosphorylated and total ERK, AKT and STAT3 (Fig. 9f). These results indicated that exogenous miR-148b-3p suppressed tumor growth in vivo through KIT.

### Downregulation of miR-148b-3p correlates with high risk grades and poor survival for GIST patients

**Fig. 10:**
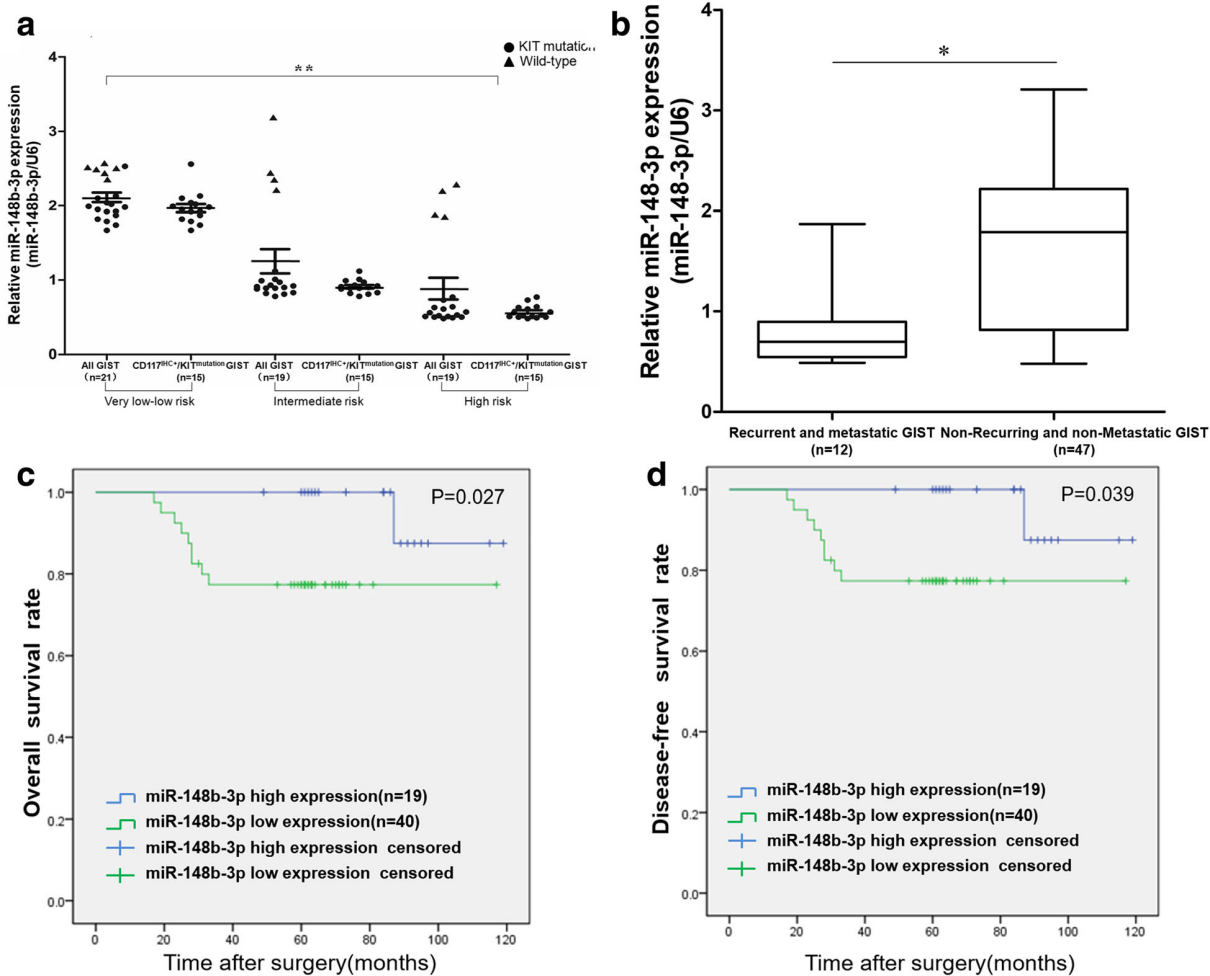
Downregulation of miR-148b-3p is correlated with high risk grades and poor survival for patients with GIST. (**a**) Relative miR-148b-3p expression levels were inversely correlated to tumor risk grade in a progressive manner. (**b**) Relative expression of miR-148-3p was significantly correlated with recurrent or metastatic GISTs. (**c**) High miR-148b-3p expression marks low OS for GIST patients. Kaplan-Meier analysis with the log-rank test for OS was performed for the 59 GIST patients whose samples were used in this work. (**d**) High miR-148b-3p expression is negatively related to disease-free survival for GIST patients. Kaplan-Meier analysis with the log-rank test for DFS was performed for the 59 GIST patients whose samples were used in this work. miRNAs expression was normalized to U6 expression. *P<0.05, **P<0.01

We identified statistically significant correlations between miR-148b-3p expression and several clinicopathological parameters, including tumor risk grade, mitotic rate and recurrence or metastasis (Table 3). Interestingly, the miR-148b-3p expression of all GIST samples as well as CD117^IHC+^/KIT^mutation^ GISTs were negatively associated with tumor risk grade in a step-wise manner (Fig. 10a). Moreover, there was a notable trend for lower miR-148b-3p expression among GIST patients with recurrence or metastasis (Fig. 10b). We also examined the relationship between miR-148b-3p expression and overall survival (OS) and disease-free survival (DFS) rates in GIST patients by Kaplan-Meier analysis with log-rank tests, and found that GIST patients with lower expression of miR-148b-3p have poorer OS rates and higher tumor recurrence rates (*P* < 0.05, Fig. 10c-d).

## Discussion

miRNAs comprise a class of small noncoding RNAs that implicated to undertake prominent roles in multiple tumor-related processes, such as oncogenesis, progression, invasion, and metastasis [9–11]. miRNA dysregulation has been established as one of the key characteristics contributing to carcinogenesis [11]. Recently, numerous studies have explored the association between miRNA expression profiles and GIST pathogenesis, which implicated that the dysregulation of miRNAs is associated with anatomical localization, mutation status, chromosome 14q loss, tumor risk, and biological behavior of GISTs [12–15, 22]. As gain-of-function mutations of KIT and KIT overexpression are characteristic features of GISTs [3], the aberrant expression of miRNAs that interrelates with KIT-induced tumorigenesis of GISTs is of particular interest [13, 22, 23].

Although the presence of KIT mutations usually leads to strong KIT expression in GISTs, the mechanism of KIT overexpression is not yet well understood [24]. Dysregulation of miRNAs expression might relevant with KIT mutations and KIT overexpression and the resulting tumorigenesis in GISTs. Recently, several studies have indicated that miRNAs can serve as critical modulators of the KIT expression, and these mechanisms are essential for the initiation and progression of GISTs. For example, Koelz et al. found the repressed expression of miR-221 and miR-222 was inversely relevant with KIT expression and might be considered as a putative molecular mechanism in the pathogenesis of GISTs [25–27]. miR-494 has been identified to downregulate KIT by directly binding to two separate seed sequences on the KIT mRNA, thereby inhibiting proliferation, migration and invasion of GIST882 cells [28]. Another microRNA, miR-218, was recently found to diminish KIT protein and suppress cell proliferation and invasion in GIST-T1 cell lines [29]. However, only a few miRNAs involved in tumorigenesis that are related to mutation of KIT have been identified in GISTs. Haller et al. assessed the relevance between miRNAs expression signatures and the mutation status in twelve GIST samples and discovered that 16 miRNAs were performanced in a mutation-dependent manner [13]. Bachet et al. also found that 13 mRNAs were specifically associated with the KIT homozygous/heterozygous mutation status in GISTs [30]. Thus, investigations on the function of miRNAs that are specifically associated with KIT mutation and KIT overexpression are required to improve the current knowledge of GIST and may offer novel insights into GIST therapy.

In this study, we evaluated the miRNA expression profiles in a cohort of CD117^IHC+^/KIT^mutation^ GISTs and CD117^IHC−^/wild-type GISTs and confirmed 6 aberrant miRNAs whose dysregulated expression was inversely related with KIT mutations and KIT overexpression. GO enrichment and KEGG pathway analyses were carried out to detect the role of abnormally expressed miRNAs in GIST pathogenesis, and the results showed that these miRNAs were obviously enriched in tumor-associated signaling pathways, including the TGF-beta signaling pathway, the MAPK signaling pathway and the Wnt signaling pathway. Among the validated miRNAs, miR-148b-3p and miR-140-5p were predicted to target KIT, PDGFRA, PIK3CA, AKT2, PTEN or EGFR genes, which are important intermediate molecules of the KIT signaling pathway as shown by the miRNA gene network analysis [19, 31]. Based on the structural analysis of the six candidate miRNA and the 3′-UTR of KIT miRNA, we found that only miR-148b-3p could directly target KIT mRNA by interacting with the 3′-UTR of KIT at two conserved putative binding sites. According to the above results, miR-148b-3p is postulated to be a tumor suppressor involved in GIST tumorigenesis by directly binding to the 3′-UTR of KIT.

Accumulating evidence has demonstrated that miR-148b-3p downregulation is a frequent event in multiple tumors, such as pancreatic cancer, breast cancer, colorectal cancer, gastric cancer and lung cancer [32–38]. Functional studies also revealed that miR-148b-3p could act as a tumor suppressor via directly targeting distinct oncogenes such as DNMT3b [33], CCK2R [34], DNMT1 [36], CEA [37] and AMPKα1 [38] in these cancers. Moreover, miR-148b-3p downregulation is responsible for the inhibition of cell proliferation, progression and invasion in gastric cancer, pancreatic cancer and NSCLC [35–38], as well as for distant metastasis or poor prognosis of breast cancer and pancreatic cancer [32, 38]. Although the underlying molecular mechanism of miR-148b-3p involved tumorigenesis has been identified in several studies, the probable role of miR-148b-3p in GISTs remained to be elucidated. Therefore, we detected the molecular mechanism of miR-148b-3p in GISTs by gain- and loss-of-function analysis. Our data revealed that miR-148b-3p upregulation dramatically suppressed proliferation, migration and invasion of GIST882 cells as well as reduced the tumorigenic ability of GIST882 cells in nude mice. Besides,miR-148b-3p interacted synergistically with imatinib in the inhibition of cell proliferation and sensitizes GIST882 cells to imatinib treatment. Clinical analysis also demonstrated that the low expression of miR-148b-3p in tumor specimens of GISTs was correlated with poor prognostic factors, including high risk grade, high mitotic rate and the presence of recurrence or metastasis. These results indicated that miR-148b-3p downregulation might contribute to the progression of GISTs, and thus miR-148b-3p could be recommended as a promising prognostic biomarker in GISTs.

Although previous bioinformatics analyses have indicated KIT as a direct target of miR-148b-3p, the dual-luciferase reporter assay was further carried out and confirmed that only miR-148b-3p could directly target KIT by binding to the potential 3′-UTR at nucleotides 1639–1656. Transfection of miR-148b-3p mimics or inhibitor was found to alter the KIT protein level without affecting its mRNA level, which suggested that miR-148b-3p negatively regulates KIT by a post-transcriptional mechanism. Furthermore, western blotting confirmed that miR-148b-3p downregulated KIT expression but that this could be restored by transfection of the cells with KIT plasmid. Using CCK-8 and transwell assays, we observed that restoration of KIT expression not only rescued the cell growth inhibition induced by miR-148b-3p but also reversed the migration and invasion suppressor functions of miR-148b-3p in GIST882 cells. Taken together, these results imply that miR-148b-3p inhibits cell proliferation, migration and invasion of GIST via directly binding to KIT.

It is generally known that GISTs with KIT mutations exhibit a high level of phosphorylated KIT and display a constitutive activation of downstream signal transduction pathways that are relevant with malignant transformation, including PI3K/Akt, Ras/ERK, and JAK/STAT [19, 31]. Thus, effects of miR-148b-3p on various signaling cascades of KIT were demonstrated in this study, showing that the downregulation of KIT induced by miR-148b-3p dramatically suppresses the expression of phosphorylated and total ERK, AKT and STAT3 in vitro and in vivo. Moreover, restoration KIT expression notably increased the expression of phosphorylated and total ERK, AKT and STAT3 in GIST cells transfection with miR-148b-3p. These results indicated that the miR-148b-3p-dependent effects on GIST tumorigenesis are mediated by oncogenic KIT-related signal transductions.

## Conclusions

Our study provides convincing evidence that miR-148b-3p is frequently downregulated in GISTs with KIT mutations and KIT overexpression and its expression is negatively relevance with poor prognosis of GISTs. Moreover, miR-148b-3p functions as a tumor suppressor in suppressing cell proliferation, migration, and invasion through directly targeting KIT, and it could be exploited as a prognostic biomarker to predict the aggressive behavior as well as a therapeutic target in GISTs.

## Abbreviations

3’-UTR: 3′-untranslated region
FDR: False discovery rate
FFPE: Formalin-fixed paraffin-embedded
GISTs: Gastrointestinal stromal tumors
GO: Gene Ontology
KEGG: enrichment and Kyoto Encyclopedia of Genes and Genomes
miRNAs: MicroRNAs
NIH: National Institute of Health
PDGFRA: Platelet-derived growth factor receptor alpha polypeptide
SD: Standard deviation
SDHB: Succinate dehydrogenase complex subunits B
WT: Wild-type
