# Correction: miR-148b-3p functions as a tumor suppressor in GISTs by directly targeting KIT

**DOI:** 10.1186/s12964-023-01435-3

**Published:** 2023-12-18

**Authors:** Yu Wang, Jun Li, Dong Kuang, Xiaoyan Wang, Yuanli Zhu, Sanpeng Xu, Yaobing Chen, Henghui Cheng, Qiu Zhao, Yaqi Duan, Guoping Wang

**Affiliations:** 1 Institute of Pathology, Tongji Hospital, Tongji Medical College, Huazhong University of Science and Technology, 1095 Jiefang Dadao, Wuhan, 430030 People’s Republic of China; 2 Department of Pathology, School of Basic Medicine, Tongji Medical College, Huazhong University of Science and Technology, Wuhan, 430030 People’s Republic of China; 3 Department of Gastroenterology, Zhongnan Hospital, Wuhan University, Wuhan, 430071 People’s Republic of China

**Correction: Cell Commun Signal 16, 16 (2018)**

**https://doi.org/10.1186/s12964-018-0228-z**

Following publication of the original article [1], the authors recently noticed that the picture of the control panel ‘mimic NC’ in the Fig. 6b of this paper was misused, which is actually from the ‘inhibitor NC’ group. This error occurred during the figure preparation, in which we need to process a large quantity of pictures to generate the figures. We sincerely apologize for this unconscious mistake. We have carefully reviewed and checked the data of this figure, believing that this error does not affect the results and the conclusions of our research. The updated figure 6 is supplied in this correction article.

**Fig. 6:**
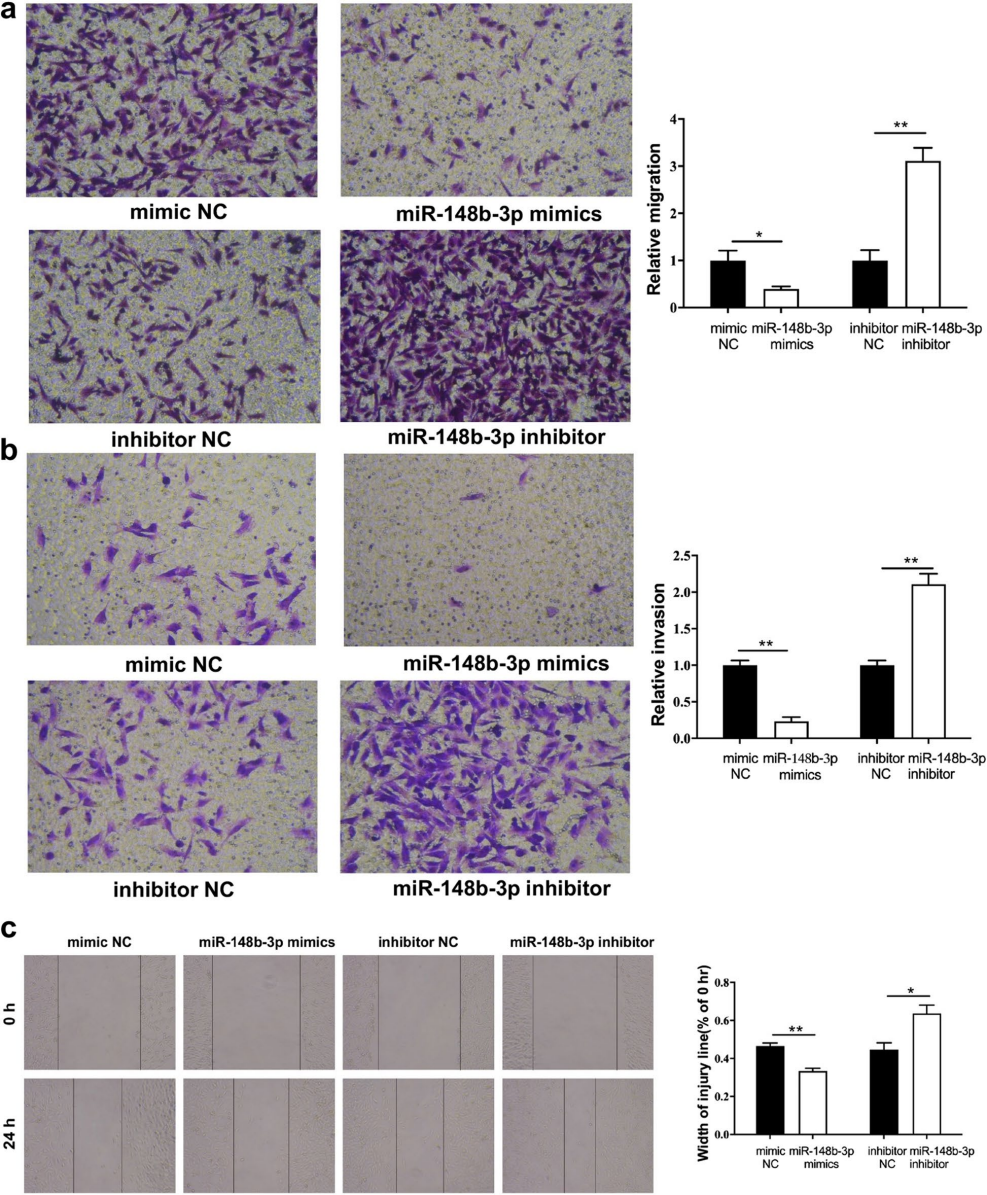
miR-148b-3p suppresses migration and invasion of GIST882 cells. (**a**) Transwell migration assay was applied to assess the migratory capacities of GIST882 cells. (**b**) Invasionassay was applied to detect the invasive capacities of GIST882 cells. (**c**) Wound healing assay was carried out to investigate the migratory ability of GIST882 cells. *P<0.05, **P<0.01
